# Parental Knowledge and Preventive Strategies in Pediatric IgE-Mediated Food Allergy—Results from a Cross-Sectional Survey

**DOI:** 10.3390/nu17152387

**Published:** 2025-07-22

**Authors:** Francesca Galletta, Angela Klain, Sara Manti, Francesca Mori, Carolina Grella, Leonardo Tomei, Antonio Andrea Senatore, Amelia Licari, Michele Miraglia del Giudice, Cristiana Indolfi

**Affiliations:** 1 Pediatric Unit, Department of Human Pathology in Adult and Developmental Age ‘Gaetano Barresi’, University of Messina, 98124 Messina, Italy; francygall.92@gmail.com; 2 Department of Woman, Child and General and Specialized Surgery, University of Campania ‘Luigi Vanvitelli’, 80138 Naples, Italy; klainangela95@gmail.com (A.K.); caro.grella94@gmail.com (C.G.); michele.miragliadelgiudice@unicampania.it (M.M.d.G.); cristianaind@hotmail.com (C.I.); 3 Allergy Unit, Meyer Children’s Hospital IRCCS, 50139 Florence, Italy; francesca.mori@meyer.it (F.M.); leonardo.tomei@unifi.it (L.T.); 4 Department of Clinical, Surgical, Diagnostic, and Pediatric Sciences, University of Pavia, 27100 Pavia, Italy; antonioandrea.senatore01@universitadipavia.it; 5 Pediatric Clinic, Fondazione IRCCS Policlinico San Matteo, 27100 Pavia, Italy; amelia.licari@unipv.it

**Keywords:** food allergy, parental knowledge, anaphylaxis management, pediatric allergy education

## Abstract

**Background/Objectives**: Food allergy (FA) is a growing concern in pediatric care, requiring effective avoidance strategies and timely emergency responses. The role of caregivers is central to the daily management of FA. This study aimed to assess parental knowledge, preparedness, and behaviors regarding pediatric FA management, focusing on both prevention and emergency readiness. **Methods**: A cross-sectional survey was conducted from December 2024 to April 2025 through the SurveyMonkey^®^ platform, promoted by the Italian Society of Pediatric Allergology and Immunology (SIAIP). The anonymous, structured questionnaire was distributed online and in two Italian university hospitals. A total of 129 fully completed responses from caregivers of children with FA were analyzed. The survey explored self-perceived knowledge, symptom recognition, preventive actions, emergency preparedness, and communication practices. **Results**: Only 9.3% of parents considered themselves “very informed,” while 54.3% reported limited or no knowledge. Just 16.0% recognized all symptoms of an allergic reaction, and only 24.0% could distinguish mild reactions from anaphylaxis. Notably, 67.4% reported not knowing how to respond to anaphylaxis, and 83.7% did not possess an epinephrine auto-injector. Preventive measures at home were inconsistently applied, and 41.1% took no precautions when eating out. Communication with external caregivers was often informal or absent. Only 33% updated physicians regularly. **Conclusions**: The findings reveal significant gaps in parental preparedness and highlight critical areas for educational intervention. Enhanced caregiver training, standardized communication protocols, and improved clinical follow-up are essential to strengthen pediatric FA management and safety.

## 1. Introduction

Food allergy (FA) is a growing public health concern, particularly in the pediatric population, where prevalence rates have significantly increased over the past two decades [1]. FA can lead to a broad range of clinical manifestations, from mild skin reactions to life-threatening anaphylaxis [2]. Management primarily relies on strict allergen avoidance and prompt administration of epinephrine in case of accidental exposure [2]. While emerging therapeutic options such as oral immunotherapy (OIT) and biologic agents have shown promise for selected patients, they remain accessible to a limited subset and require specialist supervision [2,3,4]. Consequently, the cornerstone of effective FA management in real-life settings remains the knowledge, vigilance, and preparedness of caregivers, particularly parents, who serve as primary coordinators of dietary choices, emergency preparedness, and communication with other caregivers (e.g., teachers, relatives) [5]. Therefore, parental education plays a crucial role in reducing the risk of accidental exposure and improving the outcomes of allergic reactions. However, gaps in knowledge and preventive behaviors remain a challenge [5,6]. Limited understanding of symptom severity, insufficient communication with secondary caregivers, and underuse or misuse of emergency medications such as epinephrine auto-injectors have been documented in various settings [5,6].

## 2. Materials and Methods

This study aims to assess parental knowledge and self-reported readiness in managing pediatric food allergies, using a structured and accessible questionnaire. This cross-sectional study was promoted by the “Primary and Secondary Prevention of Allergic Diseases” Committee of the Italian Society of Pediatric Allergology and Immunology (SIAIP), to assess the knowledge, attitudes, and behaviors of caregivers managing food allergies (FAs) in children and adolescents aged 0 to 18 years. The questionnaire was created and administered through the SurveyMonkey^®^ platform (Momentive Inc., San Mateo, CA, USA) and was open from December 2024 to April 2025. Participants were enrolled at their initial allergological evaluation at the pediatric allergy outpatient services of two tertiary university hospitals: AOU “G. Martino” in Messina and AOU “Luigi Vanvitelli” in Naples, Italy. Eligible subjects included children with a previously confirmed diagnosis of IgE-mediated food-induced anaphylaxis established at another healthcare institution, as well as those presenting with a clinical history strongly suggestive of such condition. In all cases, the assessment was performed by board-certified pediatric allergists in accordance with the diagnostic criteria outlined by the European Academy of Allergy and Clinical Immunology (EAACI), which describes anaphylaxis as a serious, rapidly evolving, and potentially life-threatening allergic reaction. It is a systemic hypersensitivity response that typically involves multiple organ systems, most commonly the skin, respiratory tract, and/or cardiovascular system [7]. Importantly, not all of the patients had already been prescribed an epinephrine auto-injector at the time of the visit. Participation was entirely voluntary and anonymous. No identifying data were collected. The survey consisted of ten closed-ended, multiple-choice questions, each offering three to four fixed response options. Caregivers were instructed to select a single answer for each item. The questionnaire was specifically designed to explore multiple dimensions of FA management, including caregivers’ self-perceived knowledge, their ability to recognize the main symptoms of an allergic reaction, and their understanding of the distinction between mild and severe reactions, such as anaphylaxis. Other areas of focus included strategies for allergen avoidance at home and outside the home, preventive behaviors in everyday settings, communication practices with external caregivers (such as teachers and relatives), the frequency with which medical updates are provided to healthcare professionals, knowledge of emergency procedures in the event of anaphylaxis, and the availability of an epinephrine auto-injector. Descriptive statistical analysis was carried out using Microsoft Excel^®^ (Microsoft Corporation, Redmond, WA, USA). Prevalence data were calculated for each response option to identify trends in knowledge and behavior, and to highlight areas in which additional caregiver education may be warranted.

## 3. Results

A total of 129 fully completed responses were collected.

### 3.1. Caregiver Knowledge and Perceptions

**Figure 1:**
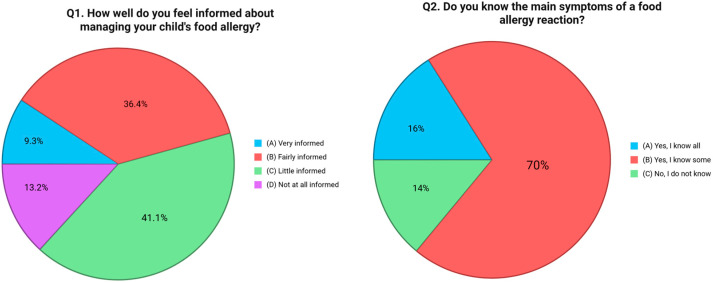
Distribution of responses to Q1 (“How well do you feel informed about managing your child’s food allergy?”) and Q2 (“Do you know the main symptoms of a food allergy reaction?”).

**Figure 2:**
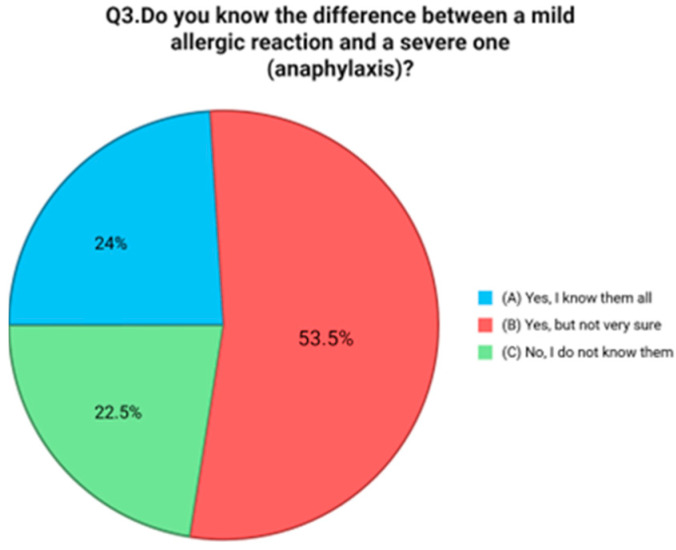
Distribution of responses to Q3 (“Do you know the difference between a mild allergic reaction and a severe one [anaphylaxis]?”).

**Figure 3:**
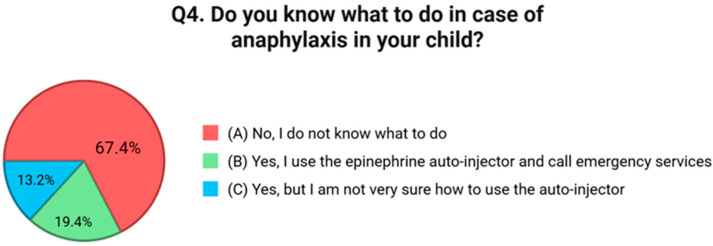
Distribution of responses to Q4 (“Do you know what to do in case of anaphylaxis in your child?”).

**Figure 4:**
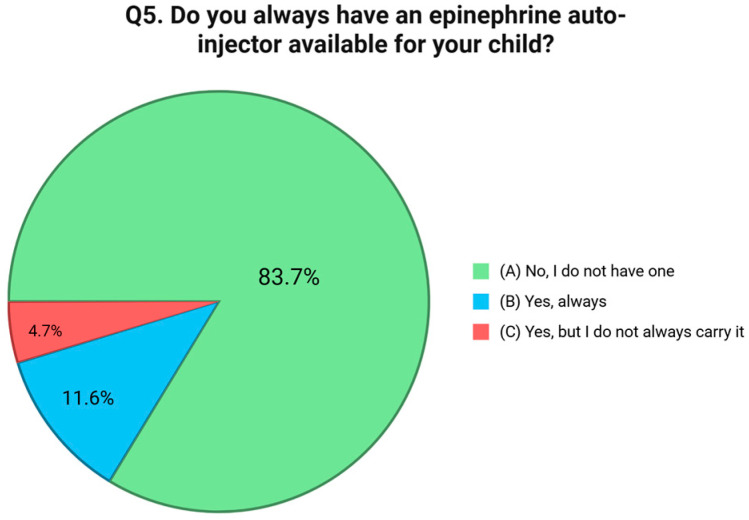
Distribution of responses to Q5 (“Do you always have an epinephrine auto-injector available for your child?”).

The first question investigated parents’ self-perceived level of knowledge in managing their child’s FA. Results showed that 41.1% of participants considered themselves “a little informed,” 36.4% “fairly informed,” while only 9.3% reported being “very informed”. Notably, 13.2% admitted to feeling “not at all informed” [Figure 1]. The second question focused on symptom recognition, a critical element of timely and effective intervention. While 70.0% of respondents reported knowing “some” of the main symptoms of a FA reaction, only 16.0% said they were familiar with all of them. Alarmingly, 14.0% acknowledged not knowing the symptoms at all [Figure 1]. The third item explored whether caregivers understood the distinction between mild allergic reactions and severe ones like anaphylaxis. While 24.0% of parents reported having a solid understanding of this difference, 53.5% stated that they had some awareness but were not confident in distinguishing the two. Furthermore, 22.5% admitted they had no knowledge on this point [Figure 2]. Question four evaluated knowledge of emergency response procedures in the case of anaphylaxis. Just 19.4% of caregivers reported knowing exactly what to do, including how to use an epinephrine auto-injector and when to call emergency services. An additional 13.2% said they were unsure how to use the auto-injector, and 67.4% admitted they would not know how to respond at all [Figure 3]. Question five focused on the availability of an epinephrine auto-injector, which represents the first-line treatment for anaphylaxis. Only 11.6% of respondents reported always having an auto-injector readily available. A further 4.7% had one but did not carry it consistently. A total of 83.7% of participants stated they did not have an epinephrine auto-injector at all [Figure 4].

### 3.2. Behavioral Practices and Risk Management

**Figure 5:**
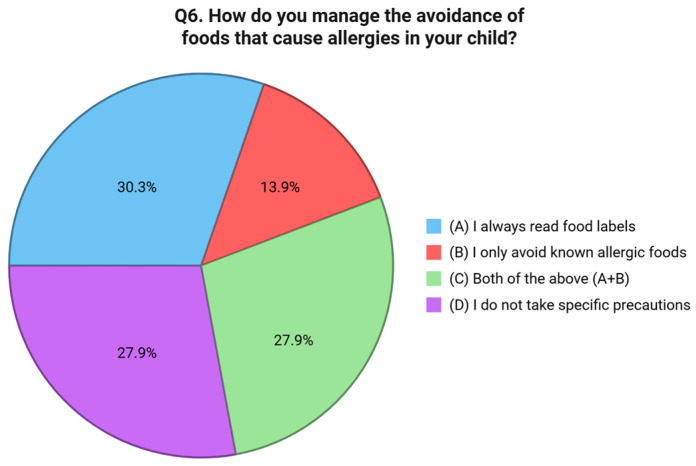
Distribution of responses to Q6 (“How do you manage the avoidance of foods that cause allergies in your child?”).

**Figure 6:**
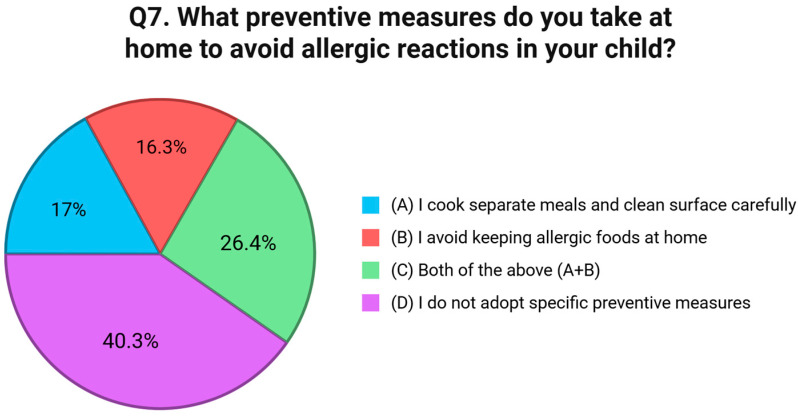
Distribution of responses to Q7 (“What preventive measures do you take at home to avoid allergic reactions in your child?”).

**Figure 7:**
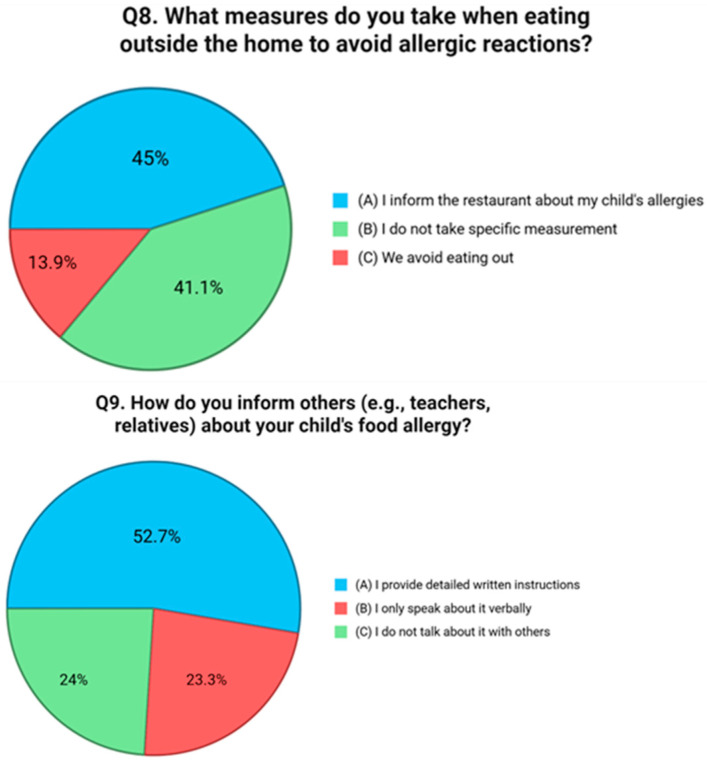
Distribution of responses to Q8 (“What measures do you take when eating outside the home to avoid allergic reactions?”) and response to Q9 (“How do you inform others [e.g., teachers, relatives] about your child’s food allergy?”).

**Figure 8:**
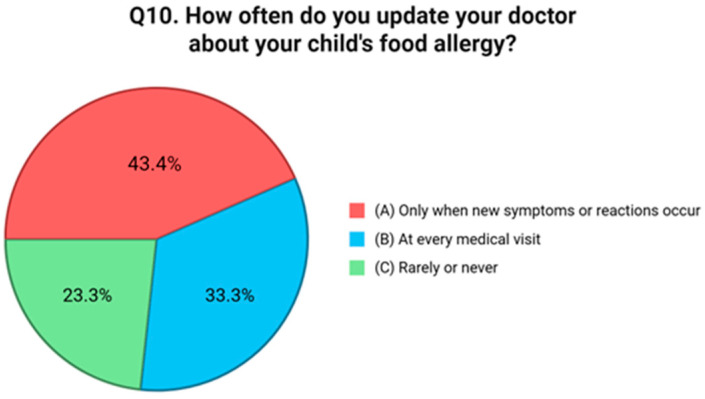
Distribution of responses to Q10 (“How often do you update your doctor about your child’s food allergy?”).

Question six addressed behavioral strategies for avoiding allergenic foods. Just 30.2% of respondents reported that they always read food labels carefully, and 13.9% said they limit avoidance to known allergens. Interestingly, 27.9% reported using both approaches, but an equal proportion, 27.9%, admitted to not adopting any specific avoidance strategies at all [Figure 5]. Preventive actions taken within the home environment were assessed in question seven. A total of 40.3% of respondents reported not adopting any targeted preventive measures. Among those who did, 17.1% stated they cook separate meals and clean surfaces carefully, while 16.3% avoid keeping allergenic foods in the home. A further 26.4% indicated that they follow both strategies [Figure 6]. The eighth question examined how families manage FA risks while eating outside the home. A total of 44.9% reported informing restaurant staff about their child’s allergy, while 13.9% said they avoid eating out entirely. However, a concerning 41.1% stated that they do not take any specific precautionary measures when dining in public venues [Figure 7]. Communication with external caregivers and institutions was the focus of question nine. Slightly more than half of the respondents (52.7%) reported providing written instructions to others about their child’s allergy. Meanwhile, 23.3% relied only on verbal communication, and 24.0% admitted that they do not inform others at all [Figure 7]. Question ten explored the frequency with which caregivers update healthcare providers about their child’s allergy status. Only 33.3% reported discussing the allergy during every medical visit. A larger group, 43.4%, stated they do so only in the event of new symptoms or allergic reactions. Notably, 23.3% reported rarely or never updating their physician unless prompted [Figure 8].

## 4. Discussion

Our survey findings highlight a complex picture of parental knowledge and behavior regarding pediatric food allergies. While a substantial proportion of parents reported being aware of allergic reactions and preventive measures, several areas of concern emerged, particularly in emergency preparedness and external communication. Despite growing public attention to food allergies, the results suggest that a large proportion of families still feel underinformed and underprepared, especially in emergency contexts. Self-perceived knowledge was generally limited: although 36.4% rated themselves as “fairly informed”, only 9.3% of respondents considered themselves “very informed” about FA management, while a combined 54.3% rated themselves as only “a little” or “not at all” informed.

These findings are consistent with prior research, which documented substantial parental knowledge gaps. Taha et al. [8], in a cross-sectional study, reported a median knowledge score of 7 out of 15 among parents of children with food allergies, with wide variability across different sections. Knowledge was significantly influenced by factors such as educational level, income, and the number of interactions with healthcare professionals, indicating substantial informational gaps within the general population [8]. Notably, most caregivers are not adequately aware of allergy symptoms, and many are unable to distinguish between a mild allergic reaction and a life-threatening anaphylactic episode. In addition, parental knowledge regarding the correct use of an epinephrine auto-injector remains limited, compromising their ability to respond effectively in emergencies. This limit is clinically significant, as delayed or incorrect administration of epinephrine is a well-documented factor contributing to the progression and severity of allergic reactions [7].

Several studies have highlighted limited practical knowledge regarding the use of epinephrine auto-injectors among caregivers [9,10,11,12]. Polloni et al. [9] highlighted a gap between parents’ self-perceived knowledge and their actual ability to manage food-induced anaphylaxis. Despite many rating their knowledge as moderate to high, only 20% had ever administered epinephrine, with widespread insecurity and fear regarding its use and side effects. These findings suggest that emotional and psychological barriers, alongside technical skills, play a critical role in emergency response [9]. Similarly, Narchi et al. [10] reported that many parents, despite having received medical training on the use of the epinephrine auto-injector, exhibited considerable hesitation when faced with real-life emergency situations. While 79% of participants were aware of the clinical indications for epinephrine use, only 36% felt competent following practical experience [9,10]. As with Polloni et al.’s findings, key barriers included fear, anxiety, and a lack of confidence in their ability to act appropriately [9,10]. To address these challenges, the authors advocate for implementing comprehensive support strategies, including structured theoretical and practical training sessions, clear and detailed written instructions, access to psychological support, and establishing peer support groups. Such multifaceted interventions may help reduce parental anxiety, enhance confidence, and ultimately improve the timely and effective use of epinephrine in anaphylactic emergencies [9,10]. This need is further confirmed by two recent cross-sectional studies, which show that parents not only continue to seek more practical guidance on when and how to administer epinephrine but also explicitly call for additional educational resources, clear step-by-step instructions, and ongoing support, including access to psychological counselling and peer groups [11,12]. In Cronin et al. [11], for example, only 26.7% of parents performed all the critical steps of the auto-injector correctly, while the Australian survey by Stockhammer et al. [12] highlighted persistent frustration and anxiety directly linked to the lack of practical training and support networks. Preventive actions within the home environment are inconsistently implemented among surveyed families. In our sample, 40.3% of respondents reported not adopting any targeted preventive measures despite the critical role of home-based strategies in minimizing allergen exposure. Among those who implemented precautions, 17.1% reported cooking separate meals and cleaning surfaces carefully, while 16.3% avoided keeping allergenic foods at home. Notably, only 26.4% of participants combined both practices, suggesting that comprehensive allergen avoidance protocols are not routinely applied.

Moen et al. [13] characterize the home as a perceived “safe zone” for families managing food allergies; however, preserving this safety requires continuous vigilance, structured routines, and careful planning. The responsibility for allergen control often falls on mothers, a role frequently associated with significant psychological burden, including anxiety and social withdrawal. In this context, a correct understanding of food labels is crucial in enabling families to identify hidden allergens and reduce the risk of accidental exposure. Strengthening parental labelling skills could significantly improve home-based food allergy management [14,15]. Another critical finding involves the management of food allergies outside the home. While many parents adopt effective avoidance strategies at home, fewer report feeling confident when dining out or leaving their child in the care of others, such as at school.

Particularly, school is where children spend most of their time. This context makes the school environment pivotal for effective food allergy management. As reported in the scoping review by Santos et al. [5], up to 20% of anaphylactic reactions occur in schools, yet preparedness remains inconsistent. Many staff members lack training in allergy and anaphylaxis management, and some are unaware of students’ food allergies. The absence of standardized policies and emergency plans is common. While educational interventions have shown benefit, the review underscores the need for mandatory training, ready access to epinephrine auto-injectors, and individualized emergency protocols to ensure student safety [5]. An important insight emerges from the analysis of parental strategies at restaurants. Although 45% of respondents reported informing restaurant staff about their child’s food allergies, this was a positive and proactive behavior. Over 41% declared they did not take any specific precautionary measures, and 13.9% explicitly stated that they avoided dining out altogether. These findings highlight a fragmented and inconsistent approach to risk management in restaurant settings. The high percentage of parents who do not adopt specific measures may reflect a combination of low-risk perception, lack of practical guidance, or skepticism about the effectiveness of communicating with food service staff. This is particularly concerning given the evidence in the literature showing that restaurants are frequent sites of accidental allergic reactions [16,17]. Furthermore, systematic reviews confirm that restaurant staff often lack adequate knowledge and training in allergen management, increasing the potential for cross-contamination and miscommunication [18,19]. The coexistence of proactive and avoidant behaviors highlights the need for targeted education and clear public health messaging to enhance caregiver preparedness in public settings. Equally important is effective communication with external caregivers, such as teachers, relatives, and family friends, which remains a key component of food allergy management. Survey results revealed substantial variability in how parents communicate information about their child’s allergy: 53% reported providing detailed written instructions, 23% relied solely on verbal explanations, and 24% admitted not informing others at all. This inconsistency is concerning, as informal or absent communication increases the likelihood of misunderstandings or errors in allergen avoidance and emergency response. These findings suggest a gap between recommended safety practices and real-world parental behavior. Clinical guidelines emphasize the importance of written documentation, including individualized emergency plans and symptom recognition tools, as essential components of safe allergy management outside the home [2,8]. As such, greater efforts are needed to promote and facilitate the use of standardized communication tools, ideally supported by healthcare professionals and institutional frameworks, to enhance safety across all caregiving environments [2,8]. The frequency with which caregivers update healthcare providers about their child’s food allergy status represents an important indicator of continuity and quality of care. In our survey, only 33% of respondents reported informing their physician at every medical visit, while a larger proportion (43%) stated they do so only when new symptoms or allergic reactions occur. Notably, 23% reported rarely or never updating their doctor unless specifically prompted.

This pattern highlights a reactive rather than proactive approach to food allergy management in the clinical setting. Regular communication with healthcare providers is essential for monitoring the child’s condition and updating emergency care plans, reviewing avoidance strategies, and evaluating the continued need for epinephrine auto-injectors or diagnostic re-assessment [20,21]. The lack of consistent dialogue may hinder timely interventions and contribute to gaps in prevention and preparedness, particularly as new triggers or changes in symptom severity can emerge over time [20,21].

### Strengths and Limitations

The main limitations of the study include the cross-sectional design and self-reported nature of the survey, which may introduce bias. However, the structured questionnaire offers a comprehensive overview of real-world behaviors and beliefs, making the findings relevant for clinical and educational policy development. Importantly, we deliberately chose not to collect individual demographic (e.g., age, sex) or clinical (e.g., specific food allergens) information about the children involved. This decision was based on multiple considerations. First, the core objective of the study was to explore parental knowledge, preparedness, and management practices at the initial clinical visit rather than to establish correlations with child-specific clinical profiles. Second, ethical and privacy concerns guided our methodology: by avoiding collecting sensitive or identifiable health data, we ensured anonymity and avoided the need for formal ethical approval or data protection procedures. Third, from a logistical perspective, maintaining a concise and non-invasive questionnaire contributed to higher completion rates and greater patient adherence, particularly in the real-life context of busy outpatient clinical settings. Lastly, as highlighted in the literature, the core principles of food allergy management, such as label reading, availability of an epinephrine auto-injector, and preparedness for emergency response, are broadly consistent across pediatric age groups and allergen types [7,9,10,11,12]. Therefore, the omission of detailed clinical variables does not compromise the relevance or validity of the study’s primary findings.

## 5. Conclusions

This study highlights several gaps in parental knowledge and preparedness in managing pediatric food allergies among patients presenting with food-induced anaphylaxis at their first clinical evaluation and initial care. While some caregivers demonstrate awareness of allergic symptoms and prevention strategies, a substantial proportion report limited understanding of anaphylaxis, uncertainty in emergency response, and inconsistent use of epinephrine auto-injectors. Communication with healthcare professionals and external caregivers is also suboptimal, potentially compromising the safety of allergic children. These findings underscore the need for comprehensive, accessible educational initiatives that promote practical skills, confidence in emergency management, and consistent communication practices for families of children with food allergies and the general population. Integrating structured parental education into routine clinical care may enhance long-term outcomes and reduce the risk of severe allergic reactions.

## Data Availability

Data are contained within the article.

## Abbreviations

The following abbreviations are used in this manuscript:

FA	Food Allergy
OIT	Oral Immunotherapy
IgE	Immunoglobulin E
FDA	Food and Drug Administration
EAACI	European Academy of Allergy and clinical Immunology
SIAIP	Italian Society of Allergy and Immunology
PTSD	Post Traumatic Stress Disorder
